# Development of a SNP-based assay for measuring genetic diversity in the Tasmanian devil insurance population

**DOI:** 10.1186/s12864-015-2020-4

**Published:** 2015-10-14

**Authors:** Belinda Wright, Katrina Morris, Catherine E. Grueber, Cali E. Willet, Rebecca Gooley, Carolyn J. Hogg, Denis O’Meally, Rodrigo Hamede, Menna Jones, Claire Wade, Katherine Belov

**Affiliations:** University of Sydney, Faculty of Veterinary Science, Rm 303, RMC Gunn Building, Sydney, NSW 2006 Australia; San Diego Zoo Global, San Diego, CA USA; Zoo and Aquarium Association, Mosman, NSW 2088 Australia; University of Tasmania, School of Biological Sciences, Hobart, Tas 7001 Australia

**Keywords:** Next-generation sequencing, Single nucleotide polymorphism, Captive breeding, Population management, Pedigree assessment

## Abstract

**Background:**

The Tasmanian devil (*Sarcophilus harrisii*) has undergone a recent, drastic population decline due to the highly contagious devil facial tumor disease. The tumor is one of only two naturally occurring transmissible cancers and is almost inevitably fatal. In 2006 a disease-free insurance population was established to ensure that the Tasmanian devil is protected from extinction. The insurance program is dependent upon preserving as much wild genetic diversity as possible to maximize the success of subsequent reintroductions to the wild. Accurate genotypic data is vital to the success of the program to ensure that loss of genetic diversity does not occur in captivity. Until recently, microsatellite markers have been used to study devil population genetics, however as genetic diversity is low in the devil and potentially decreasing in the captive population, a more sensitive genotyping assay is required.

**Methods:**

Utilising the devil reference genome and whole genome re-sequencing data, we have identified polymorphic regions for use in a custom genotyping assay. These regions were amplified using PCR and sequenced on the Illumina MiSeq platform to refine a set a markers to genotype the Tasmanian devil insurance population.

**Results:**

We have developed a set of single nucleotide polymorphic (SNP) markers, assayed by amplicon sequencing, that provide a high-throughput method for monitoring genetic diversity and assessing familial relationships among devils. To date we have used a total of 267 unique SNPs within both putatively neutral and functional loci to genotype 305 individuals in the Tasmanian devil insurance population. We have used these data to assess genetic diversity in the population as well as resolve the parentage of 21 offspring.

**Conclusions:**

Our molecular data has been incorporated with studbook management practices to provide more accurate pedigree information and to inform breeding recommendations. The assay will continue to be used to monitor the genetic diversity of the insurance population of Tasmanian devils with the aim of reducing inbreeding and maximizing success of reintroductions to the wild.

**Electronic supplementary material:**

The online version of this article (doi:10.1186/s12864-015-2020-4) contains supplementary material, which is available to authorized users.

## Background

The Tasmanian devil (*Sarcophilus harrisii*) has recently suffered a severe population decline due to the recently emerged devil facial tumour disease (DFTD) [1]. DFTD is a rare form of cancer that is transmissible, with tumour cells acting directly as the infectious agent [2]. DFTD is spread amongst devils when they bite each other during social interactions [3] with the biter, rather than the bitten, being more likely to become infected [4]. The spread of DFTD has been rapid and extensive, covering more than 85 % of the devils’ range since it was first observed in 1996 [5], with localised declines on the east coast of Tasmania of 90 % [1]. Initially it was suggested that extinction in the wild could occur within 25–30 years [1], however disease has been present for almost 20 years on the east coast without any documented evidence of localised extinction (Save the Tasmanian Devil Program, *pers. comm.*). The Tasmanian devil also suffers from low genetic diversity at both neutral and functional loci [6–8]. The devil is listed as endangered due to the disease [9] and a captive breeding program has been established to prevent extinction of the species. This insurance program has the aim of preserving 95 % of founding genetic diversity for a period of 50 years [10].

Captive breeding programs have often achieved limited success because in many cases the species of conservation concern already shows reduced genetic diversity and/or small population size. Small population size can result in a founder effect, and potentially lead to loss of fitness via inbreeding depression [11, 12]. Genetic adaptation to captivity [13, 14] and further loss of genetic diversity in captive populations [15] can occur, leading to decreased fitness upon reintroduction to the wild [16, 17]. To avoid inbreeding depression and maintain adaptive potential in captivity, breeding interactions are managed to maximize retention of genetic diversity. Traditionally this has been achieved through either pedigree management using studbooks or more recently using neutral markers such as microsatellites [15]. There is a move now to integrate studbook information and molecular data [18], and early studies have highlighted issues of using studbook-based management alone, as ancestries are often incomplete, unknown or inaccurate [19–21].

Some have questioned which types of markers are most informative to assess and manage diversity of threatened populations [22–24]. Allelic diversity is essential to the efficacy of multi-allelic markers such as microsatellites, especially at the level of individual discrimination. Loss of alleles over time in small populations may decrease the utility of such markers in captive breeding programs when it is necessary to determine relatedness between individuals. Another potential disadvantage of microsatellite markers is in their inability to make predictions of the functional consequences of loss of alleles over time in captivity [25].

Single nucleotide polymorphisms (SNPs) occur throughout the genome. SNPs are typically biallelic, and unlike microsatellites with many alleles at each locus, the number of loci is a critical consideration when assessing the power of a marker set. Between 30 and 80 SNP loci are sufficient to detect moderate to low population structure, although more loci may be needed to detect subtle population differentiation [26]. For parentage and pedigree studies 60–100 SNPs are expected to provide sufficient resolution to construct accurate pedigrees, with likelihood-based estimates being more efficient than exclusion-based methods for pedigree construction [27]. For any given population, the number of individuals sampled and number of loci genotyped must be optimised to achieve appropriate statistical power to detect a given effect [28]. As SNPs are ubiquitous in the genome and inexpensive to assay, adding further SNP loci to a marker set is relatively simple and can mitigate against the lack of allelic diversity common to small or inbred populations. SNPs are known to have a low genotyping error rate [29] and allele calling is generally more consistent than for microsatellites. These features make SNPs practical for high-throughput genotyping and data sharing across laboratories and organisations [27].

With the ever-decreasing costs of next-generation sequencing (NGS), large-scale sequencing projects in conservation programs are becoming increasingly feasible. Sequencing of PCR amplicons has benefitted greatly from high-throughput next-generation sequencing technology and it is now possible to simultaneously sequence large numbers of samples at multiple loci [30]. In species with a reference genome, it is possible to distribute markers across the genome and target protein coding regions to gain true insights of genome-wide and genic diversity.

Within the Tasmanian devil insurance population, our research group has observed that microsatellite markers have often been unable to differentiate between closely related individuals [31]. We have now taken an amplicon-based approach to target SNPs dispersed across the devil genome. We have used these target SNPs to examine genetic diversity within the Tasmanian devil insurance population and tested whether they provide more accurate discrimination of familial relationships than traditional microsatellite markers. The Tasmanian devil genome has been published [8, 32] enabling us to target genes of interest in addition to regions with putatively neutral variation. Sequencing functional regions of the genome enables us to not only assess genetic diversity and relatedness but also to make predictions about the potential functional consequences of allelic loss that may occur as a result of captive breeding.

## Results

### SNP discovery and pilot study

Re-sequencing data from 7 whole genomes were added to 3 existing genome sequences (including the reference) to form the ascertainment panel from which over six million SNPs were discovered. Using whole genome alignment of these individuals we targeted 170 SNPs within 10 loci of interest for amplicon sequencing. These included 7 putatively neutral loci, two immune genes (toll-like receptors) and one behavioral gene (dopamine receptor). After amplifying and sequencing these loci in a trial run of 20 devils, 53 SNPs were excluded as they fell below the minor allele frequency (MAF) inclusion criterion (0.03). This inclusion criterion was chosen to account for errors arising from sequencing or alignment, or alleles occurring in such a low frequency as to be unsuitable for genotyping. One amplicon provided no sequencing data and an additional 93 SNPs were identified in the trial run devils across all amplicons. Potential duplications in the immune markers led to the exclusion of these amplicons as duplications can lead to an over-estimate of heterozygosity. Four amplicons were selected from the trial run for continued use in the genotyping assay. The original trial run also identified the need for a streamlined normalization and clean up step of PCR product prior to library preparation as there was a large difference in reads produced between samples likely due to amplification bias leading from differences in input DNA (Additional file 1: Fig S1). This led to the inclusion of the SequalPrep (Invitrogen) plate-based normalization kit into the protocol.

### Genotyping of insurance population

We have genotyped 305 Tasmanian devils from the Tasmanian devil insurance population (total cohort is 693 as at July, 2015; Zoo and Aquarium Association, unpublished) using 17 different amplicons across four Illumina MiSeq runs. These 17 amplicons include four from the pilot study and an additional 13 amplicons (8 targeting immune loci and 5 putatively neutral loci). The average sequencing cover was close to 200-fold. Over 1800 SNPs were identified in total, although many of these were excluded from later analysis due to their sub-optimal MAF. Manual inspection of the excluded SNPs showed that the majority constituted errors in sequencing or alignment, or were rare (possibly de-novo) alleles that were unable to be confirmed with our data. The final genotypic dataset is derived from 17 amplicons covering 267 SNPs in regions of both putatively neutral (7 amplicons) and functional (10 amplicons) loci (Table 1; additional primer details in Additional file 1: Table S1). These 17 amplicons include three that were discontinued after the first sequencing run of 96 individuals from the insurance population (RAB27A, KIT, FOXA2) but are reported here as they provided genotypic information for those devils in the first sequencing run.

**Table 1:** Characteristics of all amplicons used in genotyping assay. Detailed results for individual SNPs within all non-immune loci included in the final assay is provided in the Additional file 1: Tables S1 and S2

Amplicon	Target variation	Chr^a^	Length (bp)	No. of SNPs	No. of SNPs not in HWE	No. of haplotypes
AGA	Neutral	6	9352	34	2 (6 %)	2
CCLD5/6^b^	Chemokine	4	8209	25	22 (88 %)	18
CX3C^b^	Chemokine	1	9425	3	1 (33 %)	6
DIG12^b^	NK receptor	3	6351	15	0 (0 %)	3
DIG24^b^	NK receptor	3	6809	1	1 (100 %)	2
DRD5	Dopamine receptor	6	7885	1	0 (0 %)	2
ERN2	Pro-apoptotic	2	10,165	17	17 (100 %)	2
FOXA2	Neutral	1	10,122	39	4 (10 %)	29
IL17B^b^	Interleukin	1	5717	20	1 (5 %)	2
IL22F1^b^	Interleukin	1	6531	3	2 (67 %)	3
KIT	Neutral	2	8356	2	1 (50 %)	3
NF2	Neutral	2	9312	41	1 (2 %)	3
PLEKHM3	Neutral	3	10,034	16	2 (13 %)	5
RAB27A	Neutral	1	10,077	2	0 (0 %)	3
TGFB1^b^	Cytokine	3	2649	2	0 (0 %)	3
TLR3^b^	Toll-like receptor	6	7663	32	4 (13 %)	6
UNC13B	Neutral	2	9687	14	0 (0 %)	3
Total			138,344	267	58	95

Nb. Amplicons are named according to nearest annotated gene: amplicons are not necessarily targeting the gene they are named for

^a^Chromosome as indicated by Tasmanian devil reference genome v7.1 [32] however recent work has identified that chromosomes 1 and 2 were mis-identified [50]

^b^Indicates immune-targeted amplicon [40]

One amplicon targeting putatively neutral loci (FOXA2) displayed a potential ascertainment bias whereby SNPs that were identified in the original ascertainment panel did not consistently amplify in all insurance population devils. This amplicon was therefore removed from further sequencing. Two amplicons (RAB27A, KIT) failed to amplify sufficient SNPs to be informative (two SNPs each) and so were also removed. All of the remaining amplicon loci, both neutral and functional (224 SNPs across 14 amplicons), were included in the assessment of devils in runs 2, 3 and 4.

A comparison of four individuals who were repeated as positive controls gave an error rate of genotype calling (after filtering) of 0 % when using neutral loci only and 7 % when immune genes were included. This error rate may indicate the presence of segmental duplication as many immune genes are known to be highly duplicated [33]. Consequently a subset of SNPs with lower reliability were removed from the analysis.

### Diversity statistics

**Fig. 1:**
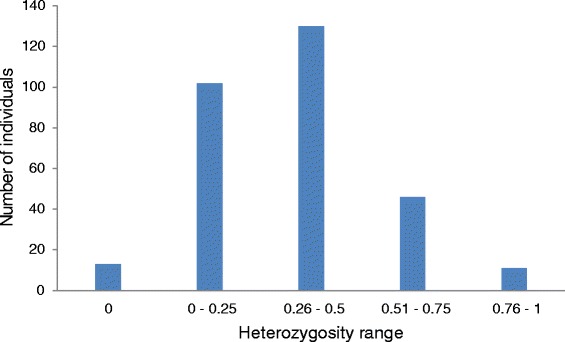
Numbers of individuals within each range of heterozygosity level for 302 Tasmanian devils within the insurance population. Three devils excluded as < 30 SNP loci successfully genotyped

There were 13 devils from the insurance population that were found to be 100 % homozygous in each of the 30–170 SNP loci that were successfully amplified in these animals (Fig. 1). Eight of these individuals were founders. Two sets of siblings were also observed to be 100 % homozygous which confounded efforts to successfully assign parentage for these offspring. Of the remaining genotyped insurance population devils, 102 had very low observed heterozygosity levels (<0.25). The majority of the population displayed generally low individual heterozygosity, though there was a wide variance in the population (Fig. 1).

The majority of SNP loci located within putatively neutral amplicons were observed to segregate in Hardy-Weinberg equilibrium (HWE) (Table 1) (see also Additional file 1: Table S2). Deviations from HWE are expected to be influenced by relatedness within the insurance population. It should be noted that some parent-offspring and full-sibling relationships are included in this dataset which violated the assumption of a homogeneous population and may have influenced estimates of heterozygosity and HWE. The numbers of observed haplotypes varied greatly across amplicons (Table 1). It is possible that the high number of haplotypes observed for FOXA2 was due to the inconsistent amplification of SNPs between individuals. As noted above, this amplicon was excluded from further sequencing. Haplotype frequencies for the remaining neutral loci are reported in the online materials (Additional file 1: Table S3).

### Pedigree analysis

To demonstrate the utility of the SNP assay we conducted an assessment of parentage assignment within the insurance population. Of the 305 devils genotyped using SNPs, 66 were offspring for whom either the sire or dam (or both) were unknown. There were an additional 10 offspring for whom parentage was known from studbook records and this parentage was confirmed using SNP data. Of the 66 offspring with unknown parentage we were able to assign parentage with confidence in 21 cases (Table 2). Of the remaining 45 offspring, it was not possible to assign parentage to 12 offspring because not all candidate parents were available to be genotyped. This meant that for these animals only predictions and/or exclusions were possible (Table 2). Predictions and exclusions were also made for the remaining 33 offspring although the definitive assignment of parentage was not possible in these cases. In two offspring, too few loci were successfully amplified to enable any assessment of parentage. The average percentage of missing genotypes across animals assessed for pedigrees was 44 % and average number of shared genotyped loci between offspring/dam/sire trios was 109. There were 7 offspring with fewer than 20 shared SNP loci amongst some trios due to a potential dam failing to amplify many SNPs. Exclusions only were provided for these offspring. Although SNP amplification failure in 3 individuals (2 offspring and 1 dam) affected our ability to assign parentage in these cases, lack of diversity amongst candidates was the key issue in our inability to assign parents in the remaining offspring.

**Table 2:** Pedigree results for all genotyped offspring

Facility/Group	No. of offspring	Potential dams	Potential sires	No of loci genotyped	Average % missing gts	Comments
1/1	2	1 known	2	164	0.51	All resolved
1/2	2	1 known	2	164	0.61	All resolved
1/3	4	1 known	4	164	0.60	One unresolved- two sires possible
1/4	4	1 known	5	164	0.60	All resolved
1/5	7	4	4	164	0.52	One unresolved- two dams and two sires possible
1/6	4	1 known	4	164	0.70	All resolved
2/1	8	5	8	192	0.22	Exclusions only- full-sibs and half sibs amongst candidate parents, 1 dam failed genotyping
2/2	13	5 known	8	192	0.24	Exclusions only- half-sibs amongst candidate sires, no sample for 1 known dam
3/1	5	3	5	192	0.29	Exclusions only- full- and half-sibs amongst candidate sires
4/1	6	3	12	192	0.26	Exclusions only- half-sibs amongst candidate sires and no sample for one candidate dam
5/1	11	8	6	192	0.28	Exclusions only- half-sibs amongst candidate parents

Nb. Number of loci differ as trios from breeding facility 1 were genotyped in the first sequencing run whereas trios from breeding facilities 2–5 were sequenced in runs 2, 3 and 4 with a refined marker set. Known dams are according to information provided from studbook management

There were eight offspring who were genotyped at both SNPs and microsatellites which allowed us to compare the relative efficacy of each method for parentage assignment. These eight offspring came from three separate breeding groups, all with a known dam and 4–5 potential sires. Sire predictions were made for each offspring using both genotyping methods but some differences were evident. Allelic diversity was low across the majority of the microsatellite markers and this limited discrimination among potential sires. For two of the eight offspring there were disagreements of predicted sire using the different genotyping methods (Additional file 1: Table S4). The higher trio LOD scores for the SNP data suggests more power to discriminate between a given male and any random males in the population, in the SNP-based genotyping assay [34]. This is also evident in the greater confidence of trio delta scores achieved from the SNP data relative to the microsatellite data (Additional file 1: Table S4). Genotypes for all individuals included in the pedigree analysis are available in the online material (Microsatellite data: Additional file 2: Table S6; SNP data: Additional file 3: Table S7).

## Discussion

In this study we have developed a novel method for the discovery and genotyping of SNPs for use in conservation applications. Our method has successfully assessed genetic diversity in the Tasmanian devil insurance population and has determined the parentage of 21 offspring for whom this was previously unknown. The assay has many benefits over other commonly used genotyping methods and is especially versatile in its applicability and scalability. In the current study we have used the assay to assess both population diversity and for parentage as this is a priority for providing breeding recommendations in the insurance program. There is further scope to use and expand on this dataset to examine the effects of captive breeding on functional loci.

The SNP assay was used to successfully assign parentage for 21 of the 54 offspring for whom all candidate parents were genotyped. For the 33 remaining offspring, a combination of factors have influenced our ability to assign parentage. All of the successfully resolved pedigrees were genotyped in the first sequencing run (164 SNP loci compared) despite this run having higher missing genotype rates (Table 2). The high missing genotype rate was likely due to poor sample quality because high quality and quantity DNA is required for long-range PCR and NGS applications. Samples which had undergone whole genome amplification prior to PCR using REPLI-g had varying levels of success in subsequent sequencing. This is most likely due to degradation and fragmentation of input DNA and highlights the need for stringent protocols in the collection and storage of samples. These potentially degraded samples were still able to be accurately assigned parents because the pool of candidate parents was relatively small (2–5) and there was sufficient diversity amongst these candidates for differentiation. This highlights the fact that while sample quality can reduce genotyping success, lack of diversity amongst devils was the key impediment in discriminating amongst individuals. In addition, the first sequencing run exposed the need for shorter amplicon lengths to account for input sample quality that cannot always be guaranteed. DNA deterioration impedes the amplification of long DNA fragments. This problem was alleviated by a move to shorter amplicons and this is reflected in the greater genotyping success seen in samples sequenced across runs 2–4 (Table 2).

In the larger free-range enclosures where the pool of potential sires and dams is greater the analysis increases in complexity. This is especially true in instances where full-siblings and half-siblings were amongst the pool of dams or sires (Table 2), which can decrease the accuracy of parentage assignment [27]. Differing management practices across institutions may also influence accuracy of pedigree assessment, as genetic diversity in the pool of candidate parents may vary, as do the numbers of individuals housed together. Low genetic diversity in devils is expected to remain a contributing factor in our ability to accurately assign parents as heterozygosity is generally low (Fig. 1) and many common haplotypes are shared amongst the insurance population (Additional file 1: Table S3). Very low allelic diversity is evident at the microsatellite loci resulting in lower confidence of predictions made for parentage in comparison to SNP loci due to decreased power in the marker set, as predicted for a likelihood approach to parentage assignment [34].

Our findings suggest that some ascertainment bias occurred during assay development, as 70 % of the individuals used for the original ascertainment panel were from the same wild population. This issue was easily overcome with subsequent sequencing and genotyping as the amplicon-based method allows for identification of additional SNPs which did not exist in the ascertainment panel. One of the key benefits of our approach is that it is robust to such biases in marker selection. As larger numbers of animals are sequenced, the assay can be adjusted to exclude those markers that are uninformative and ascertain new markers where needed.

Heterozygosity varied widely among individuals (Fig. 1). Individual heterozygosity may be influenced by historical captive breeding as relatedness of individuals, particularly of the original founders, has not always been known and any breeding of related individuals would be expected to lead to a loss of heterozygosity. Low heterozygosity levels in the population as a whole may indicate a founder effect or lack of diversity in the original founders. Some individuals did display high heterozygosity, however as the assay has been optimized to capture diversity within the devil insurance population, this may also influence observed heterozygosity levels. There is the potential to use these diversity indices to aid species conservation programs by selecting the most heterozygous individuals for release into the wild or breeding individuals to maximize heterozygosity [13]. Care must be taken to ensure that allelic diversity is also maintained when selecting individuals for breeding. More research is required to establish how individual heterozygosity may influence fitness and whether neutral or functional loci are the best proxies for this assessment. Also, although we have used genomic information to spread our markers across the genome, more research is needed to determine to what degree the current assay reflects true genome-wide diversity. Ongoing research by members of our group using whole genome re-sequencing of larger numbers of wild-caught devils will attempt to address this latter question.

The degree of relatedness among some of the founding devils of the insurance population is not certain and our inability to discriminate between some potential parents in the current study may reflect a lack of diversity in the population founding gene pool. Future research will examine diversity in the original founders as inbreeding in the insurance population may be exacerbated by either low diversity in founding devils or potentially related founders breeding where their familial relationship was unknown [31]. Low genetic diversity in the Tasmanian devil generally may continue to confound efforts to assess devil genetic diversity and relatedness. One of the key advantages of our approach is that the genotyping assay is dynamic, allowing loci to be added over time as needed to increase power and target additional genomic regions of interest.

The genotyping assay we have developed can be adapted to study variation in any genomic region of interest in a species’ conservation program. Adaptation to captivity can have detrimental effects on a species upon reintroduction to the wild [13]. Specifically, behavioral adaptation to captivity is often a result of captive breeding scenarios, with selection for more placid behavior appearing to take place [35, 36]. SNPs within a dopamine receptor (DRD5) were included in the assay as this gene has been associated with aggression and boldness in a number of species [37–39]. Unfortunately few of the target SNPs within DRD5 amplified preventing meaningful assessment of functional variation within this gene. Further research is ongoing analyzing additional genes associated with behavior to determine whether any behavioral adaptation to captivity occurs within the Tasmanian devil insurance population. Maintenance of wild behaviors is paramount as reintroductions to the wild are already taking place and an animal’s ability to explore a novel environment is key to the success of re-introduction [40]. Aggressive interactions also appear to play a part in transmission of DFTD [4]. Further functional genes involved in reproduction are being investigated for inclusion in the genotyping assay as these genes may also play an important role in captive breeding management and reintroductions to the wild.

## Conclusions

SNP-based genotyping provides an informative and reliable method of assessing genetic diversity within captive breeding programs where large numbers of individuals and relationships must be evaluated and data is to be shared across institutions. We have shown that a next-generation sequencing approach can be used to examine diversity and determine pedigrees in a captive breeding program. The SNP-based genotyping assay we have developed provided more definitive parentage results than the microsatellite markers previously used to estimate relatedness in the Tasmanian devil insurance population. Many of the offspring genotyped were able to be accurately assigned parents though a high degree of relatedness amongst candidates and low diversity in the devil confounded efforts for many offspring and highlights the need for intensive management of genetic diversity in the insurance program. Further pedigree determination is ongoing as more samples are collected and more offspring are produced within the insurance population.

The current assay includes a good representation of diversity at both neutral and functional loci across the genome. Further markers are being developed to assess immune [41], behavioral and reproductive variation; key traits to monitor in a captive breeding scenario. Quantification of existing functional diversity within these genes will enable efforts to preserve diversity, improving the captive breeding program and subsequent reintroductions to the wild. Reintroductions to a protected island offshore from Tasmania have already taken place, with other isolated areas now coming on-line. This study provides a foundation to assess both the effectiveness of the captive breeding program, and explore how selection acts on key regions of the genome in both a zoo-based setting and in the wild.

## Methods

### SNP discovery

Seven Tasmanian devils were subjected to whole genome sequencing, establishing an ascertainment panel for identification of genomic regions most likely to harbor high levels of diversity. Devil samples for whole genome sequencing were collected in the field in north-western Tasmania with approval from the University of Tasmania’s Animal Ethics Committee (A0010296) and from the Tasmanian Department of Primary Industries and Water (TFA 08211). DNA was extracted from tissue (ear biopsy) using a phenol-chloroform extraction method [42]. Tasmanian devil genomes were sequenced by the Ramaciotti Centre for Genomics at the University of New South Wales, Kensington, using Truseq library preparation kits on an Illumina HiSeq 2000. One sample per lane was used with 100 BP paired-end reads resulting in 10–15 fold coverage. Genome sequences were deposited in the European Nucleotide Archive (accession numbers: ERS682204-ERS682210). Two additional genomes (GenBank: GCA_000219685.1, [8]) were added to the ascertainment panel. Re-sequenced genomes were aligned to the Tasmanian devil reference genome assembly version 7.0, (GenBank: GCA_000189315.1, [32]) using Burrows-Wheeler Aligner [43]. Variants including SNPs were identified using SAMtools [44] using minimum base and mapping quality scores of 20. PCR duplicates, which can arise due to PCR amplification bias during library preparation, were removed using SAMtools. A local realignment around insertions and deletions was performed using GATK IndelRealigner [45] to minimize false SNP calls from misaligned sequence. Custom Perl scripts were developed to filter the SAMtools SNP calls within and between samples. SNPs were required to be covered by at least four quality reads, with at least two reads (for low cover regions) or 20 % of reads supporting the alternate base required for a call of an alternate base.

### Primer design

Alignment of whole genome sequences to the reference genome allowed us to target loci of particular interest. These included genes involved in immunity and behavior as well as putatively neutral loci. Three loci were defined as neutral as they were a large distance from any gene (NF2- 44Kb; AGA- 53Kb; FOXA2- 290Kb). Two loci (PLEKHM3 and RAB27A) were defined as putatively neutral as they included only SNPs found within non-coding regions and KIT was also considered to be putatively neutral as it was found to be a pseudogene. One additional locus (UNC13B) has also been defined as putatively neutral as all but one of the SNPs in this locus are in non-coding regions and the one exonic SNP is not predicted to be non-synonymous. All target regions were scanned for polymorphisms and primers designed to amplify these loci (Additional file 1: Table S1). Primers were designed using the program Primer 3 plus [46] and checked for homo- and heterodimers using Oligoanalyser (Integrated DNA Technologies) [47]. A BLASTN [48] of each primer sequence was performed against the devil reference genome to check for specificity (Ensembl release 77) [49]. Additional amplicons were designed according to the devil integrated cytogenetic map [50] to ensure that all chromosomes were represented in the genotyping assay. Amplicons varied in length between 7.8 and 10.1 kilobases (Kb) and number of SNPs per amplicon varied from 1 to 41 (Table 1).

### PCR amplification and sequencing

Further Tasmanian devil samples were provided by participants in the Tasmanian devil insurance population under a University of Sydney animal ethics permit (5584). DNA was extracted from blood or tissue (ear biopsy) using either DNeasy (Qiagen), Qiamp (Qiagen) or phenol-chloroform extraction protocols. DNA concentration was assessed using a Qubit (Life Technologies). Samples (*N* = 16) that had low DNA concentration (<10 ng/μL) were amplified prior to PCR using the REPLI-g kit (Qiagen) following the manufacturers guidelines. Replicates of two samples with and without amplification were tested to ensure that no bias was introduced by the amplification process.

PCR was carried out on devil genomic DNA in a total volume of 20 μl, containing 10x SequalPrep Reaction Buffer including dNTPs (Invitrogen), 10x SequalPrep Enhancers A and B (Invitrogen), 0.5 μM each primer, 0.4U DMSO and 1.8U of SequalPrep Long Polymerase (Invitrogen). PCR amplifications were performed on a BioRad T100 Thermal Cycler at the following conditions: 100 °C hot lid; 94 °C initial activation for 2 min (min); 10 cycles of 94 °C denaturation for 10 s (sec), 55 °C annealing for 30 s, 68 °C extension for 1 min/Kb followed by 30 cycles of 94 °C denaturation for 10 s, 55 °C annealing for 30 s, 68 °C extension for 1 min/Kb increasing by 20 s/cycle; and 72 °C final extension for 5 min. Correct amplification of the target product was checked by gel electrophoresis using 1 % agarose 1× TBE gel, stained with SYBR Safe DNA gel stain (Invitrogen), alongside Hyperladder I (Bioline).

We conducted an initial pilot sequencing experiment in which 10 wild and 10 captive devils were sequenced at 10 different amplicons. Amplicons were cleaned using ExoSAP (Affymetrix) and quantified using a Qubit (Life Technologies), before pooling amplicons by sample and conducting library preparation and sequencing. Data from the initial pilot experiment were not included in our final dataset. Following this initial trial, we improved on the manual normalization in four subsequent sequencing experiments by simultaneously purifying and normalizing PCR amplicons using the SequalPrep plate-based normalization kit (Invitrogen), following the manufacturer’s instructions. The resulting products contained equal concentrations of each amplicon pooled by sample. A small number (2–4) of additional amplicons were purified and normalized manually (as above); serial dilutions were performed to obtain concentrations equivalent to that produced using the SequalPrep plate-based kit, then added to each sample pool. Pooled samples were prepared for sequencing using the Nextera XT sample preparation kit (Illumina) in conjunction with the Nextera XT index kit (Illumina), multiplexing 96 samples per run. Libraries were sequenced on an Illumina MiSeq at The University of Sydney Faculty of Veterinary Science and the UNSW Ramaciotti Centre for Genomics. Overall, a total of 305 samples (including a known family trio for proof of concept, and four duplicate samples as controls) were sequenced across four MiSeq runs.

### Data analyses

**Fig. 2:**
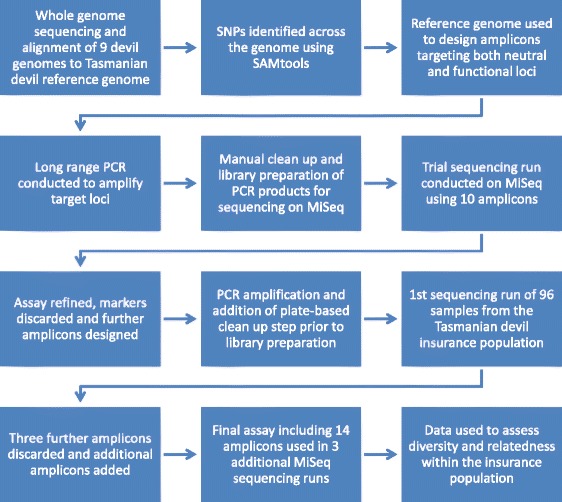
Workflow of the development of SNP-based genotyping assay

After each sequencing run, reads were separated into samples according to their unique combination of indices by the MiSeq software (Illumina), and samples were aligned separately to the devil reference genome using Burrows-Wheeler Aligner (BWA) as previously described [43]. Any sample reads that required trimming for quality were trimmed using BWA during the alignment process. SAMtools [44] was used to call SNPs with the same parameters as for the original ascertainment panel. Custom Perl scripts were employed to transform the data into PLINK v1.07 [51] format for further analyses. PLINK was used to identify those SNPs with a minor allele frequency (MAF) < 0.03 and these loci were removed from further analyses. PLINK was also used to identify loci deviating from Hardy-Weinberg equilibrium (HWE), assess heterozygosity levels and estimate haplotype frequencies for each amplicon. Default parameters were employed in all PLINK analyses. SNPs were also manually checked by viewing sequence alignments using SAMtools tview to confirm any dubious SNP calls and identify any potentially duplicated loci. We predicted whether SNPs in exons caused an amino acid change based on the devil, mouse and human gene annotations in the UCSC Genome Browser. See also Fig. 2 for an outline of the workflow of the development of the genotyping assay.

### Microsatellite markers

As a comparison with our new SNP typing approach, we compared parentage assignment for eight insurance population offspring that were also genotyped using microsatellites. Twelve existing microsatellite markers designed for the devil (six MHC linked loci [52]) and six anonymous loci [53] were used (Additional file 1: Table S5). PCR was carried out on devil genomic DNA in a total volume of 10 μl, containing 2x Type-it Multiplex PCR Master Mix including 3 mM MgCl2 (Qiagen), and 0.2 μM of each primer. PCR amplifications were performed on a BioRad T100 Thermal Cycler at the following conditions: 95 °C initial activation for 5 min; 28 cycles of 95 °C denaturation for 30 s, 60 °C annealing for 90 s, 72 °C extension for 30 s followed by a 60 °C final extension for 30 mins. PCR products were checked on a 1.2 % TBE agarose gel. The amplified products were separated by electrophoresis on an ABI 3130XL Genetic Analyser (Applied Biosystems) and scored against the size marker LIZ 500 using Genemarker v 1.95 (Soft Genetics LLC).

### Pedigree determination

The final genotyping dataset was imported into the software Cervus 3.0 [54] to assign paternity and maternity of 66 offspring for which parentage was unknown. Cervus was also used to assign paternities for the eight offspring for which microsatellite data were generated. Cervus uses a likelihood-based method of parentage assignment given allele frequencies of variable loci within the target population. Between 164 and 192 SNP loci were available for comparison in all breeding groups assessed; in the subset of individuals genotyped with microsatellites, all were successfully genotyped at the 12 loci (see Results). Allele frequencies were calculated for each breeding facility separately to account for any potential population differences across breeding facilities. Two breeding groups housed in separate pens had a known dam and two potential sires. Three breeding groups had a known dam and four potential sires. One group had five known dams across 13 offspring with eight potential sires. Five breeding groups had 3–8 potential dams and 4–12 potential sires (Table 2). These 11 breeding groups were housed across five separate breeding facilities. Due to translocation of devils some potential sires and dams are candidate parents of multiple groups of offspring.

To infer whether the microsatellite or SNP typing data gave more reliable parentage assignment, we report the trio LODs and deltas for the assigned parent, for both methods. LOD scores generated by Cervus indicate the ratio between the likelihood of parentage for an individual, relative to a randomly selected individual from the population, and incorporate genotyping error probabilities [34, 54, 55]. The highest LOD score indicates the most-likely parent [34]. In addition, Cervus calculates the difference in LOD scores (“delta”) for the two most-likely parents, and uses computational simulations of the population genotypes to evaluate a critical delta against which a confidence estimate is obtained [54, 55]. Note that LOD scores less than 0 are possible, but that only LOD scores greater than zero are used for computing delta [54, 55].

## Additional files

Additional file 1:Figure S1. Number of reads by sample in trial MiSeq run (blue bars) manually normalising across samples and amplicons using serial dilutions. Red bars indicate reads from Miseq run of samples normalised using the Sequalprep (Invitrogen) plate-based normalisation kit. Nb. Much lower density of reads/sample for those normalised using the Sequalprep kit as many more samples and additional markers included in run however coverage was far more even across samples and markers and averaged at almost 200× per sample. Trial run data was not used for any statistcial analyses. **Table S1.** Amplicon characteristics and primer details for putatively neutral loci. **Table S2.** Genomic locations, allele variants, and minor allele frequencies (MAF) for putatively neutral SNPs. Genotype refers to ratio of p^2^/2pq/q^2^. Loci showing statistically significant deviation from Hardy-Weinberg equilibrium at α = 0.05 are marked with an *. **Table S3.** Haplotypes and their frequencies for putatively neutral amplicons used in the final genotyping assay. **Table S4.** Comparison of paternity results for eight offspring genotyped at both microsatellite and SNP markers. Sire predictions which differed between the two genotyping methods are in bold. **Table S5.** Multiplex conditions for the 12 microsatellite loci genotyped herein. (DOCX 77 kb) Additional file 2: Table S6. Microsatellite genotypes for pedigree determination. (XLSX 13 kb) Additional file 3: Table S7. SNP genotypes for pedigree determination. (XLSX 279 kb)

## Abbreviations

LOD: Logarithm of the odds (to the base 10)
DFTD: Devil facial tumour disease
PCR: Polymerase chain reaction
STDP: Save the Tasmanian devil program
SNP: Single nucleotide polymorphism
NGS: Next generation sequencing
HWE: Hardy-Weinberg equilibrium
SB: Studbook
MAF: Minor allele frequency
MHC: Major histocompatibility complex
