# Supplementary material for: Development of a SNP-based assay for measuring genetic diversity in the Tasmanian devil insurance population
Source: BMC Genomics. 2015 Oct 14;16:791. doi: 10.1186/s12864-015-2020-4 (PMC4607143; doi:10.1186/s12864-015-2020-4)
Supplement: Additional file 1:Figure S1. — Number of reads by sample in trial MiSeq run (blue bars) manually normalising across samples and amplicons using serial dilutions. Red bars indicate reads from Miseq run of samples normalised using the Sequalprep (Invitrogen) plate-based normalisation kit. Nb. Much lower density of reads/sample for those normalised using the Sequalprep kit as many more samples and additional markers included in run however coverage was far more even across samples and markers and averaged at almost 200× per sample. Trial run data was not used for any statistcial analyses. Table S1. Amplicon characteristics and primer details for putatively neutral loci. Table S2. Genomic locations, allele variants, and minor allele frequencies (MAF) for putatively neutral SNPs. Genotype refers to ratio of p2/2pq/q2. Loci showing statistically significant deviation from Hardy-Weinberg equilibrium at α = 0.05 are marked with an *. Table S3. Haplotypes and their frequencies for putatively neutral amplicons used in the final genotyping assay. Table S4. Comparison of paternity results for eight offspring genotyped at both microsatellite and SNP markers. Sire predictions which differed between the two genotyping methods are in bold. Table S5. Multiplex conditions for the 12 microsatellite loci genotyped herein. (DOCX 77 kb) [file 12864_2015_2020_MOESM1_ESM.docx]

Additional file 1

Figure captions

**Figure S1** Number of reads by sample in trial MiSeq run (blue bars) manually normalising across samples and amplicons using serial dilutions. Red bars indicate reads from Miseq run of samples normalised using the Sequalprep (Invitrogen) plate-based normalisation kit. Nb. Much lower density of reads/sample for those normalised using the Sequalprep kit as many more samples and additional markers included in run however coverage was far more even across samples and markers and averaged at almost 200x per sample. Trial run data was not used for any statistcial analyses.

**Figure S1**

**Table S1** Amplicon characteristics and primer details for putatively neutral loci.

| **Amplicon** | **Length (bp)** | **No. SNPs** | **Primer sequence (5’-3’)** | **Tm^o^C** | | **Ensembl location** |  |
| --- | --- | --- | --- | --- | --- | --- | --- |
| ERN2 | 10165 | 17 | TTTCCCCCAATCTGTTCCTA | 59.4 | GL841538.1: 159977-159996 | |  |
|  |  |  | TTGCCCTTTCTAATCCCTGA | 59 | GL841538.1: 170123-170142 | |  |
| UNC13B | 9687 | 14 | GCATCCTGAGCAACCAAACT | 60.3 | GL841539.1: 2046503-2046522 | |  |
|  |  |  | TGAGTTAGCACCCTCGATACC | 59.2 | GL841539.1: 2056170-2056190 | |  |
| NF2 | 9312 | 41 | CATCCAGGTCAATCCCTGAA | 60.9 | GL842060.1:28389-28408 | | |
|  |  |  | TGCTGAGAAGAGCAAGAGCTAA | 59.7 | GL842060.1:37680-37701 | | |
| PLEK | 10034 | 16 | TCTGATAGAGAAGGGATTATTAGGAG | 57.8 | GL849899.1: 897357-897382 | | |
|  |  |  | AAGGAGCCCAGCTGAAGTTA | 59.1 | GL849899.1: 907397-907416 | | |
| AGA | 9352 | 34 | TAGGTCCGATCAAAGGCAAC | 60.1 | GL864793.1: 314413-314432 | | |
|  |  |  | GGGCAGGGAGACTCTCAATTA | 60.6 | GL864793.1: 323764-323784 | | |
| DRD5 | 7885 | 1 | GATTTTGCCACTGTGGGTCT | 60 | GL864869.1: 401583-401602 | |  |
|  |  |  | TCCCCCTGACAGTCCACTAC | 60 | GL864869.1: 409449-409468 | |  |
| RAB* | 10077 | 2 | GCAGTTATTACAAGTCATTTTGCCTA | 60 | GL834491.1:616043-616068 | |  |
|  |  |  | CCCTAAAGCATTGTATCCTTGC | 60 | GL834491.1:626099-626120 | |  |
| KIT* | 8356 | 2 | GCCTTGGAGATAATATGTGATTTG | 58.9 | GL841289.1: 742285 to 742308 | |  |
|  |  |  | TTCTCTGCCTCCTGATCCTG | 60.5 | GL841289.1: 750622 to 750641 | |  |
| FOX* | 10122 | 39 | TTGGTCCCAGTCCATTTCAT | 60.2 | GL834719.1: 21036 to 21055 | |  |
|  |  |  | ATTTGAGGGAAGTGCTGCTG | 60.4 | GL834719.1: 31139 to 31158 | |  |

***** Omitted from further sequencing runs due to either few SNPs amplifying (RAB, KIT) or apparent ascertainment bias (FOX).

**Table S2** Genomic locations, allele variants, and minor allele frequencies (MAF) for putatively neutral SNPs. Genotype refers to ratio of p^2^/2pq/q^2^. Loci showing statistically significant deviation from Hardy-Weinberg equilibrium at α = 0.05 are marked with an *.

| **Marker name** | **SNP contig position and reference allele** | **A1** | **A2** | **MAF** | **Genotype** | **O(HET)** | **E(HET)** | **P** |  |
| --- | --- | --- | --- | --- | --- | --- | --- | --- | --- |
| ERN2.3 | Chr2_0397.160216_A | T | A | 0.09 | 7/18/154 | 0.101 | 0.163 | 0.0001 | * |
|  | Chr2_0397.166864_T | C | T | 0.10 | 9/19/156 | 0.103 | 0.181 | 0.0000 | * |
|  | Chr2_0397.167259_T | T | C | 0.10 | 8/20/158 | 0.108 | 0.175 | 0.0000 | * |
|  | Chr2_0397.167436_C | C | T | 0.10 | 8/20/160 | 0.106 | 0.173 | 0.0000 | * |
|  | Chr2_0397.167456_A | G | A | 0.09 | 7/21/158 | 0.113 | 0.171 | 0.0002 | * |
|  | Chr2_0397.167603_A | G | A | 0.10 | 8/20/153 | 0.111 | 0.179 | 0.0000 | * |
|  | Chr2_0397.168015_G | A | G | 0.10 | 8/19/146 | 0.110 | 0.182 | 0.0000 | * |
|  | Chr2_0397.168022_T | C | T | 0.10 | 7/20/146 | 0.116 | 0.177 | 0.0002 | * |
|  | Chr2_0397.168607_T | T | C | 0.09 | 7/20/155 | 0.110 | 0.169 | 0.0002 | * |
|  | Chr2_0397.168661_A | A | G | 0.10 | 7/21/150 | 0.118 | 0.177 | 0.0003 | * |
|  | Chr2_0397.168755_T | T | C | 0.09 | 7/20/153 | 0.111 | 0.171 | 0.0002 | * |
|  | Chr2_0397.168785_T | T | C | 0.10 | 7/21/152 | 0.117 | 0.176 | 0.0003 | * |
|  | Chr2_0397.169005_T | C | T | 0.10 | 7/20/151 | 0.112 | 0.173 | 0.0002 | * |
|  | Chr2_0397.169068_C | C | G | 0.10 | 8/20/154 | 0.110 | 0.178 | 0.0000 | * |
|  | Chr2_0397.169525_G | A | G | 0.10 | 7/20/148 | 0.114 | 0.175 | 0.0002 | * |
|  | Chr2_0397.169609_T | C | T | 0.10 | 7/20/151 | 0.112 | 0.173 | 0.0002 | * |
|  | Chr2_0397.170050_G | G | A | 0.10 | 7/20/142 | 0.118 | 0.181 | 0.0003 | * |
| UNC13B | Chr2_0398.2046719_T | T | C | 0.45 | 52/133/80 | 0.502 | 0.494 | 0.9011 |  |
|  | Chr2_0398.2047424_G | G | A | 0.45 | 55/130/84 | 0.483 | 0.494 | 0.7125 |  |
|  | Chr2_0398.2047432_A | A | G | 0.46 | 55/138/77 | 0.511 | 0.497 | 0.7133 |  |
|  | Chr2_0398.2049841_T | T | C | 0.46 | 53/140/73 | 0.526 | 0.497 | 0.3885 |  |
|  | Chr2_0398.2050495_C | C | T | 0.46 | 56/135/78 | 0.502 | 0.497 | 0.9028 |  |
|  | Chr2_0398.2052291_G | G | C | 0.45 | 54/134/78 | 0.504 | 0.496 | 0.9016 |  |
|  | Chr2_0398.2052296_C | C | A | 0.45 | 55/132/80 | 0.494 | 0.496 | 1.0000 |  |
|  | Chr2_0398.2052757_C | T | C | 0.04 | 1/15/218 | 0.064 | 0.070 | 0.2601 |  |
|  | Chr2_0398.2053020_T | T | C | 0.46 | 55/137/76 | 0.511 | 0.497 | 0.7125 |  |
|  | Chr2_0398.2053704_G | G | A | 0.46 | 56/134/79 | 0.498 | 0.496 | 1.0000 |  |
|  | Chr2_0398.2054104_C | C | T | 0.46 | 53/138/77 | 0.515 | 0.496 | 0.6221 |  |
|  | Chr2_0398.2055158_C | C | T | 0.45 | 56/133/81 | 0.493 | 0.496 | 0.9028 |  |
|  | Chr2_0398.2055543_G | G | T | 0.47 | 55/140/72 | 0.524 | 0.498 | 0.4607 |  |
|  | Chr2_0398.2055897_C | C | G | 0.45 | 54/134/78 | 0.504 | 0.496 | 0.9016 |  |
| NF2 | Chr2_0919.28530_T | C | T | 0.26 | 18/91/131 | 0.379 | 0.389 | 0.7398 |  |
|  | Chr2_0919.28659_G | T | G | 0.24 | 14/87/137 | 0.366 | 0.367 | 1.0000 |  |
|  | Chr2_0919.28793_T | C | T | 0.24 | 15/86/141 | 0.355 | 0.365 | 0.7242 |  |
|  | Chr2_0919.28859_G | C | G | 0.25 | 16/92/136 | 0.377 | 0.379 | 1.0000 |  |
|  | Chr2_0919.28860_G | A | G | 0.25 | 16/91/137 | 0.373 | 0.377 | 0.8656 |  |
|  | Chr2_0919.29023_A | G | A | 0.26 | 18/91/135 | 0.373 | 0.385 | 0.6193 |  |
|  | Chr2_0919.29195_C | T | C | 0.26 | 17/92/137 | 0.374 | 0.381 | 0.7400 |  |
|  | Chr2_0919.29212_A | G | A | 0.26 | 19/90/136 | 0.367 | 0.386 | 0.5070 |  |
|  | Chr2_0919.29213_C | T | C | 0.26 | 20/89/136 | 0.363 | 0.388 | 0.3242 |  |
|  | Chr2_0919.29327_T | C | T | 0.25 | 16/91/138 | 0.371 | 0.376 | 0.8652 |  |
|  | Chr2_0919.29357_T | C | T | 0.25 | 16/91/137 | 0.373 | 0.377 | 0.8656 |  |
|  | Chr2_0919.29471_C | T | C | 0.25 | 17/86/137 | 0.358 | 0.375 | 0.4924 |  |
|  | Chr2_0919.29576_A | G | A | 0.25 | 16/88/138 | 0.364 | 0.373 | 0.7302 |  |
|  | Chr2_0919.29580_T | A | T | 0.25 | 16/88/138 | 0.364 | 0.373 | 0.7302 |  |
|  | Chr2_0919.29620_T | C | T | 0.25 | 17/91/139 | 0.368 | 0.378 | 0.7361 |  |
|  | Chr2_0919.29725_A | C | A | 0.26 | 19/90/138 | 0.364 | 0.384 | 0.4102 |  |
|  | Chr2_0919.29823_C | A | C | 0.25 | 15/92/139 | 0.374 | 0.373 | 1.0000 |  |
|  | Chr2_0919.30018_G | A | G | 0.26 | 18/89/134 | 0.369 | 0.384 | 0.6143 |  |
|  | Chr2_0919.30338_C | T | C | 0.25 | 15/90/139 | 0.369 | 0.371 | 1.0000 |  |
|  | Chr2_0919.30497_C | T | C | 0.25 | 15/86/131 | 0.371 | 0.375 | 0.8614 |  |
|  | Chr2_0919.30845_T | G | T | 0.26 | 17/91/134 | 0.376 | 0.383 | 0.7394 |  |
|  | Chr2_0919.31193_G | A | G | 0.25 | 15/76/123 | 0.355 | 0.373 | 0.4673 |  |
|  | Chr2_0919.31355_T | C | T | 0.05 | 3/16/193 | 0.075 | 0.098 | 0.0120 | * |
|  | Chr2_0919.34312_G | A | G | 0.26 | 18/91/133 | 0.376 | 0.387 | 0.6218 |  |
|  | Chr2_0919.34895_A | G | A | 0.26 | 19/91/134 | 0.373 | 0.389 | 0.5123 |  |
|  | Chr2_0919.34921_C | T | C | 0.26 | 18/89/137 | 0.365 | 0.381 | 0.5030 |  |
|  | Chr2_0919.34958_T | C | T | 0.26 | 19/89/135 | 0.366 | 0.386 | 0.4093 |  |
|  | Chr2_0919.34960_G | T | G | 0.26 | 17/91/135 | 0.375 | 0.382 | 0.7385 |  |
|  | Chr2_0919.35096_C | T | C | 0.25 | 16/92/140 | 0.371 | 0.375 | 0.8659 |  |
|  | Chr2_0919.35132_G | A | G | 0.26 | 20/90/140 | 0.360 | 0.385 | 0.3241 |  |
|  | Chr2_0919.35281_A | G | A | 0.26 | 18/91/140 | 0.366 | 0.380 | 0.5096 |  |
|  | Chr2_0919.35282_C | G | C | 0.26 | 18/91/140 | 0.366 | 0.380 | 0.5096 |  |
|  | Chr2_0919.35287_T | C | T | 0.25 | 18/91/141 | 0.364 | 0.379 | 0.5079 |  |
|  | Chr2_0919.35499_A | C | A | 0.25 | 18/91/144 | 0.360 | 0.376 | 0.5040 |  |
|  | Chr2_0919.36121_G | C | G | 0.25 | 18/86/137 | 0.357 | 0.378 | 0.3948 |  |
|  | Chr2_0919.36169_A | G | A | 0.25 | 16/89/136 | 0.369 | 0.376 | 0.7348 |  |
|  | Chr2_0919.36205_T | C | T | 0.26 | 17/92/134 | 0.379 | 0.384 | 0.8673 |  |
|  | Chr2_0919.36792_C | A | C | 0.26 | 19/93/144 | 0.363 | 0.381 | 0.5107 |  |
|  | Chr2_0919.37297_C | T | C | 0.25 | 18/96/147 | 0.368 | 0.378 | 0.6268 |  |
|  | Chr2_0919.37335_G | A | G | 0.25 | 18/95/146 | 0.367 | 0.378 | 0.6235 |  |
|  | Chr2_0919.37386_G | C | G | 0.25 | 19/93/145 | 0.362 | 0.380 | 0.5104 |  |
| PLEK | Chr3_0377.897441_T | C | T | 0.20 | 05-11-36 | 0.212 | 0.322 | 0.0206 | * |
|  | Chr3_0377.898554_A | G | A | 0.19 | 5/15/45 | 0.231 | 0.311 | 0.0454 | * |
|  | Chr3_0377.898628_A | G | A | 0.18 | 4/15/44 | 0.238 | 0.298 | 0.1067 |  |
|  | Chr3_0377.900375_T | G | T | 0.20 | 4/22/50 | 0.290 | 0.317 | 0.4685 |  |
|  | Chr3_0377.900444_C | A | C | 0.20 | 5/17/45 | 0.254 | 0.322 | 0.1179 |  |
|  | Chr3_0377.902176_T | C | T | 0.19 | 3/16/40 | 0.271 | 0.303 | 0.3969 |  |
|  | Chr3_0377.902281_T | G | T | 0.08 | 01-06-42 | 0.122 | 0.150 | 0.2664 |  |
|  | Chr3_0377.902727_C | A | C | 0.22 | 3/15/30 | 0.313 | 0.342 | 0.6694 |  |
|  | Chr3_0377.902993_A | G | A | 0.23 | 6/27/50 | 0.325 | 0.360 | 0.3683 |  |
|  | Chr3_0377.903361_G | A | G | 0.19 | 4/19/47 | 0.271 | 0.311 | 0.2621 |  |
|  | Chr3_0377.903493_C | T | C | 0.16 | 2/15/43 | 0.250 | 0.267 | 0.6259 |  |
|  | Chr3_0377.903494_A | G | A | 0.16 | 2/15/43 | 0.250 | 0.267 | 0.6259 |  |
|  | Chr3_0377.905701_C | T | C | 0.17 | 06-12-51 | 0.174 | 0.287 | 0.0033 | * |
|  | Chr3_0377.905962_T | C | T | 0.20 | 4/26/53 | 0.313 | 0.326 | 0.7368 |  |
|  | Chr3_0377.906104_C | T | C | 0.22 | 6/25/55 | 0.291 | 0.338 | 0.2034 |  |
|  | Chr3_0377.907337_G | A | G | 0.18 | 03-10-31 | 0.227 | 0.298 | 0.1251 |  |
| AGA | Chr6_0069.314505_A | G | A | 0.46 | 48/88/63 | 0.442 | 0.497 | 0.1185 |  |
|  | Chr6_0069.316170_C | C | T | 0.44 | 47/102/72 | 0.462 | 0.494 | 0.3415 |  |
| Ϯ | Chr6_0069.316606_C | C | T | 0.45 | 49/100/70 | 0.457 | 0.495 | 0.2749 |  |
|  | Chr6_0069.317401_C | C | G | 0.44 | 45/89/69 | 0.438 | 0.493 | 0.1183 |  |
|  | Chr6_0069.317456_A | A | C | 0.43 | 44/90/72 | 0.437 | 0.491 | 0.1192 |  |
|  | Chr6_0069.317725_T | T | G | 0.44 | 46/93/73 | 0.439 | 0.492 | 0.1247 |  |
|  | Chr6_0069.317800_T | C | T | 0.47 | 51/101/66 | 0.463 | 0.498 | 0.3405 |  |
|  | Chr6_0069.317863_T | T | C | 0.43 | 46/99/78 | 0.444 | 0.490 | 0.1720 |  |
|  | Chr6_0069.318002_G | A | G | 0.46 | 53/107/70 | 0.465 | 0.497 | 0.3534 |  |
|  | Chr6_0069.318369_T | T | C | 0.45 | 47/97/66 | 0.462 | 0.496 | 0.3312 |  |
|  | Chr6_0069.318467_T | C | T | 0.47 | 53/98/65 | 0.454 | 0.499 | 0.2186 |  |
|  | Chr6_0069.318495_G | G | A | 0.45 | 49/94/71 | 0.439 | 0.495 | 0.0991 |  |
|  | Chr6_0069.318612_C | C | T | 0.45 | 47/97/69 | 0.455 | 0.495 | 0.2678 |  |
|  | Chr6_0069.318730_A | G | A | 0.48 | 50/94/59 | 0.463 | 0.499 | 0.3252 |  |
|  | Chr6_0069.318906_G | G | A | 0.43 | 42/92/69 | 0.453 | 0.491 | 0.3165 |  |
|  | Chr6_0069.319090_T | T | C | 0.44 | 46/93/72 | 0.441 | 0.492 | 0.1260 |  |
|  | Chr6_0069.319144_G | A | G | 0.46 | 52/92/71 | 0.428 | 0.496 | 0.0537 |  |
|  | Chr6_0069.319248_A | A | C | 0.45 | 49/93/69 | 0.441 | 0.496 | 0.1259 |  |
|  | Chr6_0069.319483_G | G | A | 0.44 | 44/91/69 | 0.446 | 0.493 | 0.2004 |  |
|  | Chr6_0069.319502_A | A | G | 0.45 | 47/97/67 | 0.460 | 0.496 | 0.3304 |  |
|  | Chr6_0069.320299_T | C | T | 0.47 | 52/101/66 | 0.461 | 0.498 | 0.2792 |  |
|  | Chr6_0069.320529_C | T | C | 0.48 | 56/101/67 | 0.451 | 0.499 | 0.1798 |  |
|  | Chr6_0069.320698_T | G | T | 0.48 | 57/97/67 | 0.439 | 0.499 | 0.0797 |  |
|  | Chr6_0069.320726_G | G | A | 0.45 | 47/98/69 | 0.458 | 0.495 | 0.2719 |  |
|  | Chr6_0069.321559_G | G | T | 0.44 | 45/100/72 | 0.461 | 0.492 | 0.3377 |  |
|  | Chr6_0069.322081_G | G | A | 0.44 | 51/101/77 | 0.441 | 0.494 | 0.1092 |  |
|  | Chr6_0069.322087_G | G | A | 0.45 | 50/103/74 | 0.454 | 0.494 | 0.2275 |  |
|  | Chr6_0069.322108_C | T | C | 0.49 | 60/100/65 | 0.444 | 0.500 | 0.1091 |  |
|  | Chr6_0069.322208_A | A | G | 0.45 | 52/102/76 | 0.444 | 0.495 | 0.1418 |  |
|  | Chr6_0069.322361_G | A | G | 0.48 | 57/102/67 | 0.451 | 0.499 | 0.1453 |  |
|  | Chr6_0069.322763_T | T | A | 0.45 | 54/106/76 | 0.449 | 0.496 | 0.1501 |  |
|  | Chr6_0069.322830_A | A | G | 0.44 | 52/105/79 | 0.445 | 0.494 | 0.1464 |  |
|  | Chr6_0069.322834_G | C | G | 0.47 | 58/106/70 | 0.453 | 0.499 | 0.1893 |  |
|  | Chr6_0069.323484_G | A | G | 0.46 | 57/104/75 | 0.441 | 0.497 | 0.0886 |  |
| DRD5 | Chr6_0145.405679_A | A | G | 0.25 | 18/69/124 | 0.327 | 0.374 | 0.0678 |  |

Ϯ SNP is predicted to be within a regulatory region.

**Table S3** Haplotypes and their frequencies for putatively neutral amplicons used in the final genotyping assay.

| **Amplicon** | **Haplotype** | **Frequency** |
| --- | --- | --- |
| ERN2.3 | TCTCGGACTATTCCACG | 0.10 |
|  | ATCTAAGTCGCCTGGTA | 0.90 |
| UNC13B | CAGCTCACCATTTG | 0.55 |
|  | TGATCGCCTGCCGC | 0.42 |
|  | TGATCGCTTGCCGC | 0.03 |
| NF2 | CTCCAGTGTCCTGACCAATTGATAGTCTTAGGCCCGCATAC | 0.24 |
|  | TGTGGACACTTCATTACGCCTGTGACTGCGACTAGATCCGG | 0.71 |
|  | TGTGGACACTTCATTACGCCTGCGACTGCGACTAGATCCGG | 0.05 |
| PLEK | TAATCTTCAGCACTCG | 0.80 |
|  | TAATCTGCAGCACTCA | 0.01 |
|  | TAATCTGCAGCACTCG | 0.05 |
|  | CGGGACTAGATGTCTA | 0.13 |
|  | CGGGACGAGATGTCTA | 0.01 |
| AGA | ATTGCGTCGCTATAACGCAGTCTATAACGGAGGG | 0.57 |
|  | GCCCATCTATCGCGGTAAGACTGGGGGTAATACA | 0.43 |
| DRD5.1 | A | 0.25 |
|  | G | 0.75 |

**Table S4** Comparison of paternity results for eight offspring genotyped at both microsatellite and SNP markers. Sire predictions which differed between the two genotyping methods are in bold.

|  |  |  | Microsatelllites | | |  | SNPs | | |  |
| --- | --- | --- | --- | --- | --- | --- | --- | --- | --- | --- |
| Breeding group | Offspring | Dam (known) | No. of loci/alleles | Predicted Sire | Top trio LOD | Trio delta | No of loci/alleles | Predicted Sire | Top trio LOD | Trio delta |
| 1 | 1282 | 1034 | 12/32 | **1039** | 7.67 | 7.67^*^ | 110/220 | **1025** | 15.48 | 15.48^*^ |
| 2 | 1284 | 980 | 12/31 | 1037 | 1.06 | 1.07^+^ | 111/222 | 1037 | 43.39 | 13.78^*^ |
|  | 1285 | 980 | 12/31 | 1037 | 1.67 | 5.61^*^ | 109/218 | 1037 | 26.96 | 26.96^*^ |
|  | 1287 | 980 | 12/31 | 1037 | 1.49 | 4.85^*^ | 111/222 | 1037 | 55.78 | 55.78^*^ |
| 3 | 1295 | 1033 | 12/33 | **799** | 4.71 | 0.61^+^ | 55/110 | **1025** | 23.00 | 14.02^*^ |
|  | 1296 | 1033 | 12/33 | 1025 | -5.10 |  | 27/54 | 1025 | 11.66 | 11.66^*^ |
|  | 1297 | 1033 | 12/33 | 1025 | -6.75 |  | 37/74 | 1025 | 7.35 | 7.35^*^ |
|  | 1298 | 1033 | 12/33 | 1025 | -5.75 |  | 42/84 | 1025 | 24.60 | 13.88^*^ |

Nb. The number of loci and alleles varied between breeding groups due to differing levels of variation (microsatellites) and missing data (microsatellites and SNPs). The number of SNP loci for offspring 1295, 1296, 1297 and 1298 was sub-optimal although increased confidence in the predicted sire called is indicated by higher trio delta scores relative to microsatellites data. Trio deltas are left blank where these are undefined (all possible trio LODs were negative [Marshall et al. 1998]).

* Indicates a 95% confidence level as indicated by Cervus; + Indicates an 80% confidence level.

**Table S5** Multiplex conditions for the 12 microsatellite loci genotyped herein.

| Plex ID | Locus | Fluorophore | Reference |
| --- | --- | --- | --- |
| Plex1 | MHCI01 | NED | (Cheng and Belov 2012) |
|  | MHCI02 | PET | (Cheng and Belov 2012) |
|  | MHCI07 | NED | (Cheng and Belov 2012) |
|  | Sh2p | 6-FAM | (Jones et al. 2003) |
| Plex2 | Sh2g | 6-FAM | (Jones et al. 2003) |
|  | Sh2v | PET | (Jones et al. 2003) |
|  | Sh3o | 6-FAM | (Jones et al. 2003) |
|  | Sh6e | NED | (Jones et al. 2003) |
| Plex3 | MHCI05 | 6-FAM | (Cheng and Belov 2012) |
|  | MHCI06 | NED | (Cheng and Belov 2012) |
|  | MHCI08 | PET | (Cheng and Belov 2012) |
|  | Sh2L | VIC | (Jones et al. 2003) |
